# A new pharmacokinetic model for ^90^Y-ibritumomab tiuxetan based on 3-dimensional dosimetry

**DOI:** 10.1038/s41598-018-33160-0

**Published:** 2018-10-05

**Authors:** F. Morschhauser, B. Dekyndt, C. Baillet, C. Barthélémy, E. Malek, J. Fulcrand, P. Bigot, D. Huglo, B. Décaudin, N. Simon, P. Odou

**Affiliations:** 1 Univ. Lille, CHU Lille, EA 7365 – GRITA – Groupe de Recherche sur les formes Injectables et les Technologies Associees, Lille, France; 2 Haematology Department, Hôpital Claude Huriez, CHU Lille, F-59000 Lille, France; 3 Pharmacy Institute, CHU Lille, F-59000 Lille, France; 4 Nuclear Medicine department, Hôpital Claude Huriez, CHU Lille, F-59000 Lille, France

## Abstract

Monoclonal antibodies (mAbs) are key components in several therapies for cancer and inflammatory diseases but current knowledge of their clinical pharmacokinetics and distribution in human tissues remains incomplete. Consequently, optimal dosing and scheduling in clinics are affected. With sequential radiolabeled mAb-based imaging, radiation dosing in tissues/organs can be calculated to provide a better assessment of mAb concentrations in tissues. This is the first pharmacokinetic model of ^90^Y-Ibritumomab tiuxetan (^90^Y-IT) in humans to be described, based on three-dimensional (3D) dosimetry using single-photon emission computed-tomography coupled with computed-tomography. 19 patients with follicular lymphoma were treated initially with ^90^Y-IT in the FIZZ trial. Based on a compartmental approach individualising the vascular compartment within studied organs, this study proposes a reliable pharmacokinetic (PK) five-compartment model replacing the currently used two-compartment model and constitutes a new direction for further research. This model provides exchange constants between the different tissues, Area Under the Curve of ^111^In-IT in blood (AUC) and Mean Residence Time (MRT) that have not been reported so far for IT. Finally, the elimination process appears to occur in a compartment other than the liver or the spleen and suggests the metabolism of mAbs may take place mainly on the vascular compartment level.

## Introduction

Monoclonal antibodies (mAbs) whether alone or coupled with radioistopes or cytotoxic drugs^1,2^ are key components in therapies for many cancers and inflammatory diseases. In spite of their widespread clinical use, literature on mAb clinical pharmacokinetics (PK) remains sparse and little is known about mAb distribution in tissues^3–5^, which considerably complicates the defining of optimal mAb dosing and scheduling in clinical practice. Non-compartmental analysis, the most common approach to analysing PK data in drug development, requires a large amount of data and samples per individual to obtain precise PK parameter estimations and is inadequate for studying mAb behaviour^6^. With compartmental analysis, The mAb standard PK model is a two compartment model (central and peripheral), which can assess mAb kinetics in blood but not their distribution in tissues. Fronton *et al*.^7^ showed that this model is not compliant with current knowledge. Sequential imaging of radiolabeled mAbs *in vivo* means that the absorbed radiation dose as well as mAb concentrations can be calculated^8–11^. Radioimmunotherapy (RIT) - a targeted therapy using monoclonal antibodies (mAbs) directed to tumor-associated antigens to deliver irradiation from radionucleides to the tumor - is therefore a particularly attractive tool to modelise mAb PK in patients. For many years, two-dimensional (2D) imaging (planar whole-body scintigraphy (anterior and posterior views))^12^ has been the method of choice for dosimetric studies in RIT despite significant uncertainties in organ volume measurements affecting the accuracy of dosimetric estimates. Nowadays, it is possible to obtain more accurate radiation dose estimation in tissues/organs with three-dimensional (3D) dosimetry using single-photon emission computed-tomography coupled with computed-tomography (SPECT-CT)^13–16^, or Positron Emission Tomography coupled with computed-tomography (PET-CT)^17,18^.

Accurate mAb PK modeling requires individualised estimations of antibody concentrations in the vascular compartment within each organ. This individualising appears feasible with dosimetric studies^19^ but has not been applied in 3D dosimetric studies so far.

Yttrium 90 - Ibritumomab tiuxetan (Y90-IT) is a drug consisting of a murine anti-CD20 antibody (ibritumomab) linked to a chelator (tiuxetan) radiolabeled with 90-Yttrium (YTRACIS, Curium Pharma) according to the method described in the Zevalin monograph (ZEVALIN, Spectrum Pharmaceuticals BV) for therapy or 111-Indium for imaging (Indium 111 Chlorure, Curium Pharma) according to the method described by Ferrer *et al*.^20^. It has been approved for the treatment of follicular lymphoma (FL) in case of relapse or refractory disease or to consolidate first-line therapy. The recently reported FIZZ Study showed that fractionated RIT is effective as initial treatment for advanced-stage FL in patients with a high tumor burden^21^. Following the protocol, a subset of FIZZ patients underwent a patient-specific 3D dosimetric study.

This is the first description of a pharmacokinetic model of ^90^Y-Ibritumomab tiuxetan in humans, based on dosimetric and 3D-imaging data obtained from FIZZ patients, coupled with a pharmacokinetic compartmental approach and individualising the vascular compartment within the organs studied. This method allows describing all compartments in which mAb was measured.

## Results

### Patients

3D imaging data after the first fraction (F1) of RIT was available for 19 patients and after the 2 fractions (F1 and F2) for 13 patients since the second fraction was cancelled for 6 patients due to hematologic toxicity after F1 or the development of HAMA. Baseline characteristics are listed in Table 1. Only tumor volume is significantly different between F1 and F2 (6751 and 1850 mm^3^ respectively).

**Table 1:** Patient characteristics at baseline. *Mean (range).

		F1	F2	p
Patients		19	13	/
Gender ratio (M/F)		5/14	3/10	0.885
Age* (years)		58.8 (28–80)	59.6 (28–80)	0.954
Weight* (kg)		71.2 (49–100)	72.9 (49–83)	0.744
Height* (cm)		165.2 (157–185)	164.8 (157–185)	0.832
Body mass index* (kg.m^−2^)		26.0 (17.6–32.5)	26.8 (17.6–32.5)	0.658
Body Surface Area* (m^2^)		1.82 (1.49–2.28)	1.83 (1.51–2.28)	0.803
Tumor volume* (mm^3^)		6751 (150–25855)	1850 (0–8859)	**0.001**
FLIPI Score	Low Risk	6	4	0.907
	Intermediate Risk	4	3	
	High Risk	9	6	

### Concentration-time profiles

Estimated blood volumes of lumbar vertebrae L2-L4 (8.15 mL), liver (485 mL) and spleen (81.5 mL) are shown in Table 2.

**Table 2:** Volume of blood estimated in each organ. *Mean (Standard deviation).

Area	Lumbar vertebrae L2-L4	Liver	Spleen
Blood in corresponding volume* (mL)	8.15 (2.81)	485 (174)	81.5 (53.7)
Blood in mL.g^−1^ of organ*	0.15 (0.03)	0.29 (0.05)	0.28 (0.04)

**Figure 1:**
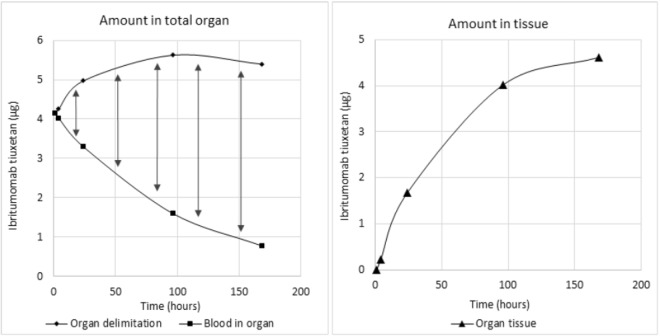
Subtraction of the blood constituent of Ibritumomab tiuxetan from total organ to obtain the remaining tissue constituent

**Figure 2:**
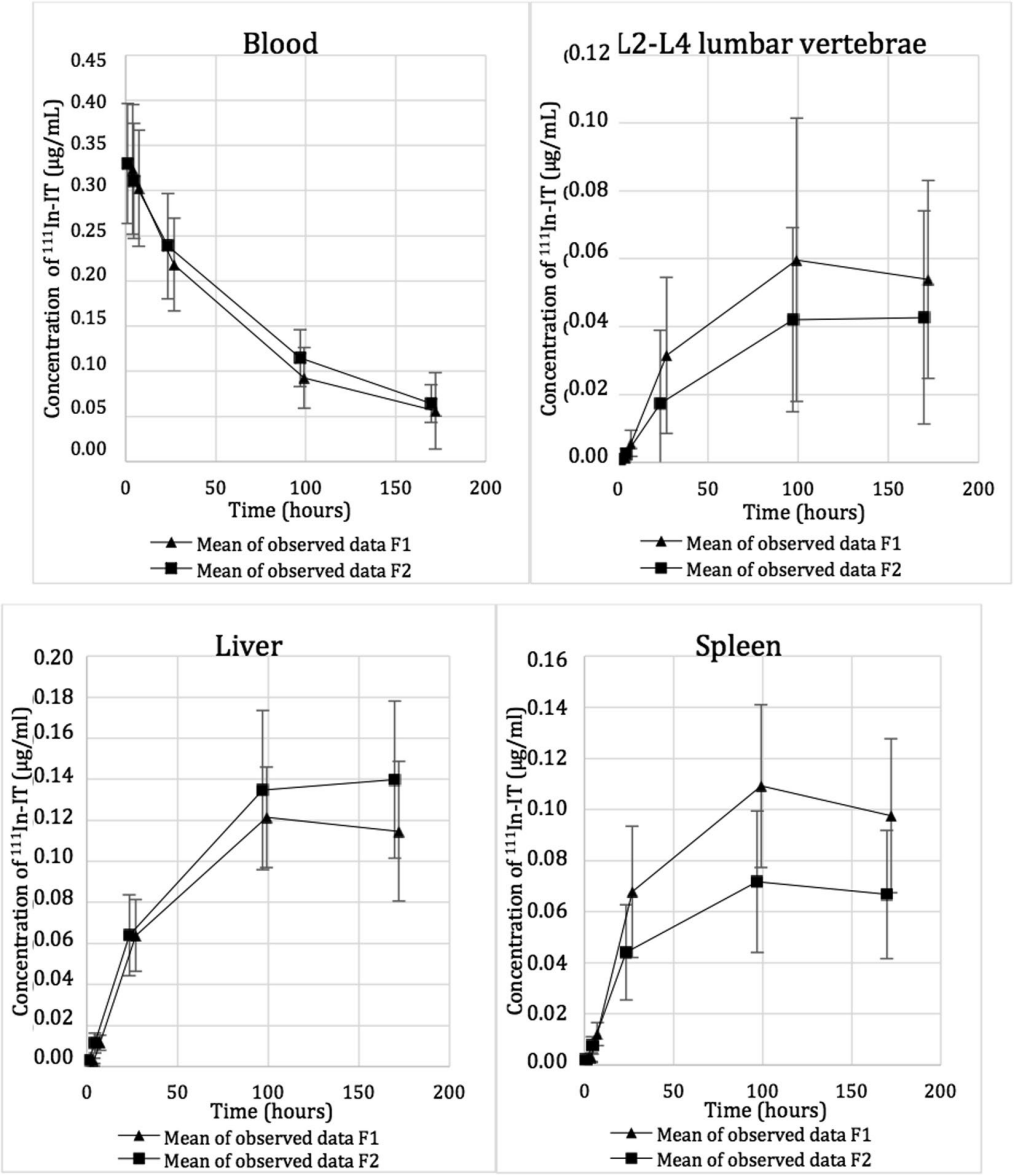
Evolution of ^111^In-IT concentration in blood and organ tissues.

The calculation method for mAb amount in blood vessels within organs and tissues is shown in Fig. 1. Radiolabeled mAb concentrations in blood and organs (concentration-time profiles) are reported in Fig. 2.

### Pharmacokinetic model

**Figure 3:**
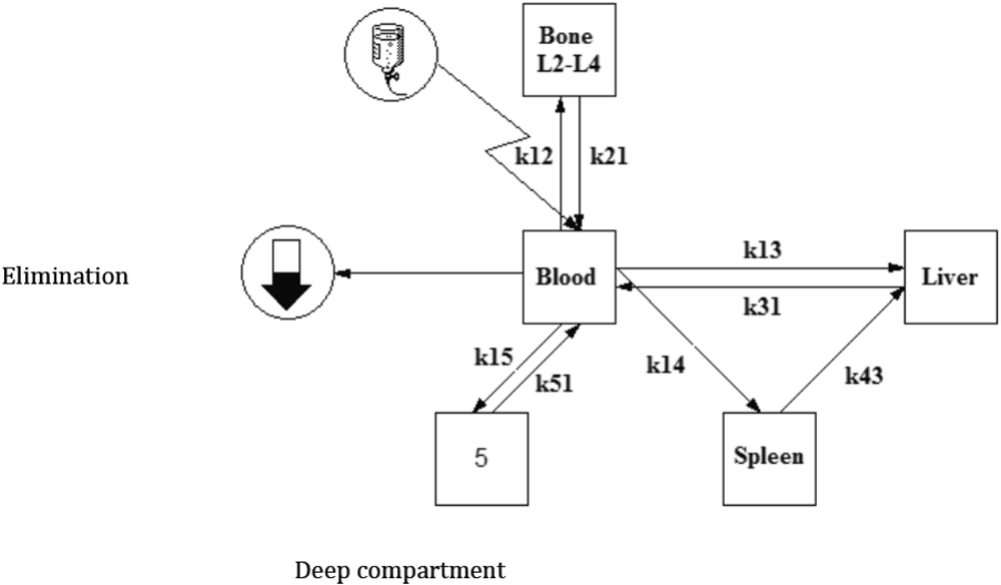
Model selected for best biodistribution fit.

The model selected with Kinetica® software to best predict the biodistribution of Ibritumomab tiuxetan is shown in Fig. 3. This model is a 5-compartment model (including blood, bone-vertebrae L2-L4, liver, spleen plus a deep compartment). In this model, ibritumomab tiuxetan is infused into blood. The vascular compartment is considered as the central compartment, from which mAbs diffuse in both directions to other compartments, except for the spleen. Radiolabeled mAbs diffuse into the spleen from the blood and are cleared by the liver. The pharmacokinetic constants are order one constants. The deep compartment (number 5) includes the remaining organs and the tumor.

### Pharmacokinetic parameters

The pharmacokinetic parameters for the various compartments (blood, L2-L4 vertebrae, liver and spleen) from Kinetica® software are reported in Table 3. Some parameters are significantly different from F1 to F2, especially for the vertebrae L1-L2 (the distribution constant k_12_ and the AUCcum 7d* (µg.min.mL^−1^) are higher for F1 than F2) and Spleen compartments (the Cmax, the distribution constant, the AUCcum 4d and AUCcum 7d are higher for F1 than F2 and the elimination constant is lower for F1 than F2).

**Table 3:** Comparison of PK parameters in F1 and F2. *Mean (Standard Deviation).

		F1	F2	p
**Blood**	Vd* (mL)	4131.0 (937.1)	4064.8 (970.1)	0.568
	Cmax* (µg.mL^−1^)	0.308 (0.086)	0.326 (0.064)	0.539
	T1/2* (h)	83.6 (37.5)	83.8 (13.5)	0.34
	MRT* (h)	114.1 (57.0)	116.0 (17.0)	0.250
	Clearance* (ml.min)	1.03 (0.73)	0.74 (0.25)	0.179
	AUC Total* (µg.min.mL^−1^)	1708.1 (820.0)	1983.7 (588.7)	0.179
**Vertebrae L2-L4**	Volume of blood* (mL)	7.84 (2.44)	7.87 (2.21)	0.944
	Cmax* (µg.mL^−1^)	0.071 (0.066)	0.046 (0.032)	0.099
	k_12_* (min^−1^)	1.11 E-4 (1.31 E-4)	0.60 E-4 (0.55 E-4)	**0.033**
	k_21_* (min^−1^)	1.05 E-4 (0.55 E-4)	0.96 E-4 (0.57 E-4)	0.516
	MRT* (h)	424.8 (136.5)	519.3 (227.2)	0.296
	AUCcum 4d* (µg.min.mL^−1^)	261.98 (251.16)	145.11 (113.73)	0.054
	AUCcum 7d* (µg.min.mL^−1^)	558.03 (530.77)	329.24 (249.35)	**0.039**
**Liver**	Volume of blood* (mL)	489.61 (178.26)	459.72 (149.07)	0.558
	Cmax* (µg.mL^−1^)	0.182 (0.126)	0.359 (0.159)	0.103
	k_13_* (min^−1^)	1.80 E-4 (0.60 E-4)	2.05 E-4 (1.70 E-4)	0.738
	k_31_* (min^−1^)	2.32 E-4 (1.07 E-4)	1.77 E-4 (0.25 E-4)	0.167
	MRT* (h)	381.4 (99.9)	419.6 (120.1)	0.357
	AUCcum 4d* (µg.min.mL^−1^)	449.72 (99.34)	548.59 (270.41)	0.277
	AUCcum 7d* (µg.min.mL^−1^)	979.63 (216.09)	1220.50 (583.78)	0.132
**Spleen**	Volume of blood* (mL)	81.22 (55.34)	65.28 (45.96)	0.295
	Cmax* (µg.mL^−1^)	0.125 (0.059)	0.073 (0.027)	**0.001**
	k13* (min^−1^)	2.11 E-4 (1.39 E-4)	1.17 E-4 (0.52 E-4)	**0.005**
	k31* (min^−1^)	1.26 E-4 (0.50 E-4)	1.64 E-4 (0.40 E-4)	**0.045**
	MRT* (h)	375.9 (119.1)	392.3 (125.5)	0.802
	AUCcum 4d* (µg.min.mL^−1^)	471.81 (230.56)	286.67 (113.86)	**0.008**
	AUCcum 7d* (µg.min.mL^−1^)	980.15 (465.22)	588.20 (225.95)	**0.004**

## Discussion

Among the thirty pharmacokinetic models assessed, the best pharmacokinetic model according to the AKAIKE test, validated on both RIT fractions, is a five-compartment model close to a mammalian PK model, except for the spleen. It provides exchange constants between the different tissues, AUC and MRT that have not yet been reported for Ibritumomab tiuxetan. Very little data is available on the elimination pathways of therapeutic mAbs. In our model, which individualises the vascular compartment within organs, the elimination process appears to be performed by a compartment other than that of the liver or the spleen and suggests the metabolism of mAbs may take place mainly on the vascular compartment level. This finding is in keeping with published data suggesting that monoclonal antibodies may be metabolised in the vascular compartment by endothelial cells and, to a much lesser extent (5.9%) urinary excretion^22–27^. Other studies, investigating the exact amount of urinary excretion through urinary sampling could provide further details on elimination pathways. However, biliary and renal excretions are known to be minimal^22–25^.

The pharmacokinetic parameters of the model concord with data in literature, thereby confirming its reliability. The mean blood half-life of ^111^In-ibritumomab tiuxetan is 83.6 and 83.8 hours for F1 and F2, respectively and varies between 20 and 140 hours in the literature^24,28^. The volume of blood in the liver (489.61 mL and 459.72 mL for F1 and F2 respectively) and in the spleen (81.22 mL and 65.28 mL) are in keeping with previously published estimates^25^. The Volumes of distribution (Vd) estimated by our model are 4131.0 and 4064.3 mL for F1 and F2, respectively. These figures are slightly lower than published data (5000–10000 mL^29^). Nevertheless the calculated volumes of distribution (our results and published data) remain small, suggesting a limited distribution outside the blood^29,30^ (29,30)(28,29). These results can be accounted for by mAb properties such as high molecular mass and hydrophilicity/polarity ratio. Other parameters cannot be compared with scientific literature as they are rarely described.

Although our 5-compartment model is a step forward compared to the previous 2-compartment model, there are clear limitations. First, in dosimetric studies, the data used to delineate organs is usually obtained with few samples: three^31,32^, four^30^, five^24,33,34^ or even fewer^35^ but this reduced sampling can prove limiting for pharmacokinetic studies. The number of image acquisitions was four (4 hours, 1, 4 and 7 days after infusions) The extrapolation method to determine the amount of blood in organs would have been more precise if imaging had been performed 1 hour after infusion, instead of 4 hours but this is problematic given the possible occurrence of infusion-related reactions to mAb. Also, the dosimetric study was limited to 7 days (taking into account the radioactive period of Indium 111 which is 67 hours). The presence of residual blood activity prevented a precise calculation of the elimination constants since the completed diffusion phase could not be assessed. It has to be acknowledged that 4 samples is already considerable as a whole body SPECT/CT examination is a time/device-consuming procedure (about 1 hour per image) and an inconvenience to patients.

A second limitation is that the compartment representing the tumor was not individualised. Tumor volume was significantly lower before F2 than F1 (1850 and 6751 mm^3^ respectively), as a consequence of tumor reduction after the first RIT fraction^36^. It should be noted that mAb concentrations were higher in blood and liver and lower in the spleen and lumbar vertebrae at F2 with significant changes in the PK parameters of “L2-L4 vertebrae” and “spleen”. This may be explained by tumor regression during the time between F1 and F2 which would modify the fifth compartment. Another study has revealed baseline metabolic tumor volume influence on mAb exposure^37^. Evaluating radiolabeled mAb distribution in the tumor compartment is difficult and tedious, in particular for small tumors. PET imaging would yield more precise quantifications due to its better spatial resolution than SPECT and to its more elaborate quantification tools. Major progress has been made in immunoPET since this study was first designed with the development of Zr89 labelled mAbs resulting in PET biodistribution studies^38,39^. It should therefore be easier to estimate the volume of tumoral lesions and, their evolution as well as modifications in radiolabeled mAb kinetics from one fraction to the other. A study has shown that CD20 expression can be induced by low dose gamma-radiation which could account for the difference in antibody distribution between the F1 and F2 fractions^40^. Another explanation could be metabolisation by the internalising^41^ of surface CD20 receptors following their association with an anti-CD20 antibody or CD20 modulation^42^.

The PK parameters described in this study are numerous and relevant and constitute a background for further research. The study proposes a reliable pharmacokinetic five-compartment model of ^90^Y-Ibritumomab tiuxetan. The main limitation to the model concerns the tumor compartment, which is not separated from the rest of the tissues. Differences in dosimetry and pharmacokinetic parameters (radiolabeled mAb concentrations, diffusion and elimination) for certain tissues might be better clarified with further investigations based on immunoPET and broader biological sampling.

## Material and Methods

### Patients

Patients with histologically confirmed CD20-positive Follicular Lymphoma were included in the FIZZ study (NCT01493479 and Northwest Ethical Research Comittee IV)^36^.

**Figure 4:**
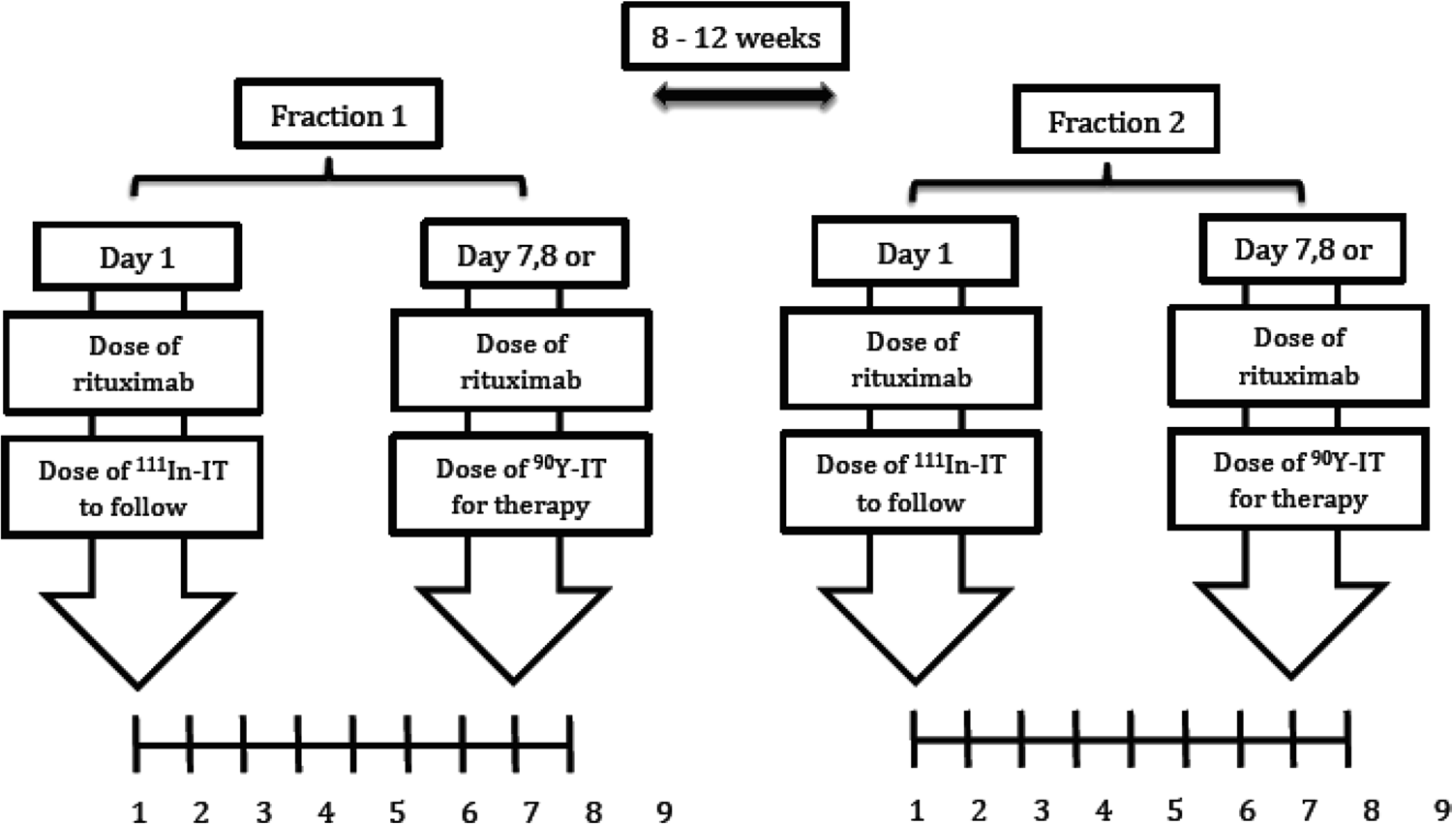
FIZZ trial design.

The FIZZ study was a multi-centre, non-randomised prospective phase II study of fractionated ^90^Y-Ibritumomab tiuxetan as the initial therapy for follicular lymphoma (FL) in patients in need of treatment according to GELF/BNLI criteria. Briefly, treatment consisted of 2 infusions, termed fraction 1 (F1) and 2 (F2) of ^90^Y-ibritumomab tiuxetan (11.1 MBq/kg per fraction; maximal dose 888 MBq) given 8–12 weeks apart (Fig. 4) provided the platelet count was >150 × 10^9^/L (2^nd^ infusion) and neutrophil count >1.5 × 10^9^/L. Occurrence of grade 3 or grade 4 myelotoxicity after 2 weeks, or a Human anti-mouse antibody (HAMA) reaction led to exclusion from a second ^90^Y-ibritumomab tiuxetan infusion. Patients with >20% lymphoma infiltration of bone marrow (BM) received 4 weekly infusions of Rituximab (375 mg/m^2^) (RTX) and proceeded to fractionated RIT only if a repeat BM biopsy demonstrated clearing of lymphoma to <20% involvement. Each ^90^Y-ibritumomab tiuxetan fraction was preceded by two rituximab infusions (250 mg/m^2^) given 7–8 days apart, the second infusion given immediately prior to ^90^Y-ibritumomab tiuxetan in order to saturate the binding sites of circulating anti-CD20 antibodies.

### Dosimetric study

Raw data used to establish a pharmacokinetic model of 90Y-Ibritumomab tiuxetan (IT) was obtained from the patients included in the FIZZ trial at Lille university hospital.

Following protocol, a subset of FIZZ patients underwent a patient-specific 3D dosimetric study with 138 MBq of ^111^In-ibritumomab tiuxetan (^111^In–ibritumomab tiuxetan administered intravenously concomitantly with the first rituximab infusion) for both 90Y- ibritumomab tiuxetan fractions^21^.

### Radiolabeling

Radiolabeling was performed with 222 MBq of ^111^In for 1.6 mg ibritumomab tiuxetan (specific activity of 138 MBq/mg). The radiochemical purity threshold for injection to the patient was set at 95%.

### Imaging method

Whole body Single-photon emission computed- tomography (SPECT)/Computed-Tomography (CT) (MEAP colimaters, Siemens medical solution, Symbia T2, Erlangem, Germany) was performed at 4 hours, 24 h, 96 h and 168 h after infusions. A CT scan was performed to delineate the organs of interest (liver, spleen, bone marrow) plus tumor volume. Concentration of radioactivity in each of these organs was determined on SPECT imaging by the count number in the respective volumes^21^. The absorbed bone marrow dose was assessed on the L2-L4 lumbar area according to Shen *et al*.^43^.

The accurate processing of 3D images to confirm absorbed-dose calculations (using CT images to estimate patient-specific organ and bone marrow volumes) was centralised in the CRCNA laboratory (Nantes) as previously^21^. All patients provided informed written consent. Ethics approval was granted in accordance with French and UK Medical Research Council guidelines and the Declaration of Helsinki (NCT01493479). All methods were performed in accordance with the relevant guidelines and regulations.

### Blood samples study

#### Blood vessel compartment in each organ

Antibody diffusion was assumed to consist of a vascular phase (from a few minutes until one hour after injection) and a tissue phase. After one hour, when antibody concentration in blood is maximal and before the tissue phase, the blood volume in each organ was extrapolated, by dividing the amount of ^111^In-IT in the whole organ (whole body SPECT/CT performed 4 hours after injection) by the concentration of ^111^In-IT in peripheral blood (sample taken 1 h after injection). Organ mass was determined using the CT scan.

#### Determination of amounts of radiolabeled mAbs in blood

The amount of radioelement in peripheral blood was calculated through decay correction and correspondence between mass and activity (activity concentration). Blood radioactivity was determined using a well counter (Packard Cobra Gamma counter, GMI Inc, Minnesota, USA) (efficiency = 93.3%). Radiolabeled antibodies were considered to be the only source of radioactivity in blood samples. The amount of ^111^In-ibritumomab tiuxetan in blood was then calculated as follows.

$$A{m}_{ibr}=\frac{Ac/0.933}{{{e}}^{-\frac{\mathrm{ln}({\rm{2}})}{{{\rm{T}}}_{1/2}^{{\rm{in}}}}.t}\times MAc}$$

Where:

- $A{m}_{ibr}$ is the amount of ^111^In-Ibritumomab tiuxetan, expressed in milligrams in the blood sample.
- $Ac$ is the detected radioactivity, expressed in MBq.
- ${T}_{1/2}^{in}$ is the radioactive half-life of ^111^In.
- $MAc$ is the activity concentration. Here, the value was 138 MBq/mg.

At each sampling time, amounts of radiolabeled mAbs in blood in organs were calculated as follows:

$$A{m}_{{ibr}{Blood}{in}{organ}}=A{m}_{{ibr}}\times {V}_{{Blood}{in}{the}{organ}}$$

#### Determination of amounts of radiolabeled mAbs in tissues

The amount of mAbs in each tissue was determined by substracting the amount of radiolabeled mAbs in blood from the total amount of radiolabeleld mAbs in organs at each sampling time.

### Pharmacokinetic model building and Modelling

The Designer module of Kinetica® software v5.0 (Thermo Fisher Scientific) was used to estimate the PK parameters of Ibritumomab tiuxetan from radiolabeled-mAb blood and organ concentrations. The quality of PK models was assessed by the Akaike Information Criterion (AIC). The best AIC score (43) indicated the most accurate model to describe PK data. The model was built from pharmacokinetic parameters which were evaluated for each patient.

These are expressed as

- kx_1_x_2_ being the order one rate constant from the x_1_ compartment to the x_2_ compartment.

- x_1_, x_2_, x_3_, x_4_ and x_5_ respectively represent blood, L2-L4 vertebrae, liver, spleen and a deep compartment, considered as the other compartments of the organism (including tumor volume).
- k_el_ is the elimination constant rate from the blood compartment (order one).
- V_d_ is the distribution volume (V_d_).
- T_1/2_ is the mAb biological half-life.
- CL_T_ is the total clearance.
- AUC is the total Area Under the Curve of ^111^In-Ibritumomab tiuxetan in blood.

Organ blood volumes were measured. The cumulated AUC at four (AUCcum 4d) and seven (AUCcum 7d) days after injection of ^111^In-Ibritumomab tiuxetan was calculated.

For each compartment, the Mean Residence Time (MRT) and maximal concentration (Cmax) of ^111^In-Ibritumomab tiuxetan were calculated.

### Statistical analysis

Data obtained after F1 and F2 was not pooled and the pharmacokinetic model was first established on data obtained after F1, followed by that obtained after F2. Parameters are expressed as mean values and standard deviation or mean values and range of variation. They were compared using the bilateral non-parametric Mann–Whitney test (α = 0.05) with XLSTAT® software (version 2012.2.03, Addinsoft).
